# Association of leukocyte mitochondrial DNA content with glioma risk: evidence from a Chinese case–control study

**DOI:** 10.1186/1471-2407-14-680

**Published:** 2014-09-19

**Authors:** Jie Zhang, Deyang Li, Falin Qu, Yibing Chen, Gang Li, Hequn Jiang, Xiaojun Huang, Hushan Yang, Jinliang Xing

**Affiliations:** Department of Oncology, the First affiliated Hospital of Chengdu Medical College, Chengdu, 610500 China; State Key Laboratory of Cancer Biology & Experimental Teaching Center of Basic Medicine, Fourth Military Medical University, Xi’an, 710032 China; Department of Neurosurgery, Tangdu Hospital, Fourth Military Medical University, Xi’an, 710032 China; Division of Population Science, Department of Medical Oncology, Kimmel Cancer Center, Thomas Jefferson University, 19107 Philadelphia, PA USA

**Keywords:** Case–control study, Mitochondrial DNA content, Peripheral blood leukocyte, Real-time PCR, Glioma risk

## Abstract

**Background:**

Increasing evidence suggests that alterations in mitochondrial DNA (mtDNA) content may be implicated in the tumorigenesis of several malignancies. However, the association between mtDNA content in peripheral blood lymphocytes (PBLs) and glioma risk has not been investigated.

**Methods:**

Real-time PCR was used to examine the mtDNA content in PBLs of 414 glioma patients and 414 matched controls in a hospital-based case–control study. The association between mtDNA content and glioma risk was evaluated using an unconditional multivariate logistic regression model.

**Results:**

We found that glioma patients exhibited a significantly higher median mtDNA content than healthy controls (0.99 vs. 0.71, *P* < 0.001). Unconditional multivariate logistic regression analysis adjusting for age, gender, smoking status, and family cancer history showed that there was an S-shaped association between mtDNA content and glioma risk. Higher mtDNA content was significantly associated with an elevated risk of glioma. Compared with the first quartile, the odds ratio (95% confidence interval) for subjects in the second, third, and fourth quartiles of mtDNA content were 0.90 (0.52-1.53), 3.38 (2.15-5.31), and 5.81 (3.74-9.03), respectively (*P* for nonlinearity = 0.009). Stratified analysis showed that the association between mtDNA content and glioma risk was not modulated by major host characteristics.

**Conclusions:**

Our findings demonstrate for the first time that a higher mtDNA content in PBLs is associated with an elevated risk of glioma, which warrants further investigation in larger populations.

## Background

Glioma is the most common primary brain tumor in both adults and children [1]. It is histologically classified into four grades (grades I-IV) according to the World Health Organization (WHO) guidelines and about 70% of glioma is malignant (grade III/IV). The key features of malignant glioma include local invasive growth and strong angiogenesis. Despite many advances in surgical and medical therapies in recent years, the clinical outcome of this disease is still dismal under the best available treatment regimen. The median overall survival is 12 ~ 14 months in glioblastoma patients and 2 ~ 5 years in anaplastic astrocytoma patients. Currently, brain-imaging technology such as magnetic resonance imaging has proven to be the most effective method of diagnosing glioma. However, the use of brain imaging is dramatically limited in early preventive screening of glioma due to its high cost and low sensitivity for early stage glioma. Although numerous genetic and molecular research projects have been focused on the development of glioma, the pathogenesis of glioma is still poorly understood. Hence, there is a pressing need to develop novel specific susceptible biomarkers for the prediction of glioma risk and early diagnosis.

Mitochondria play pivotal roles in cellular energy production, free radical generation, apoptosis, and are the major intracellular source and primary target of reactive oxygen species (ROS) [2]. Human mitochondrial DNA (mtDNA) is a circular double-stranded DNA molecule with a length of 16569 bp. Each mitochondrion contains 2–10 mtDNA molecules. The copy number of mtDNA generally remains within a relatively stable range in order to maintain the cell’s energy demands and preserve its normal physiological functions. However, mtDNA copy number may change from 10^2^ to 10^4^ per cell depending on the cell energy demands, and also vary in different cell types and tissue origins [3]. Compared with nuclear DNA, mtDNA lacks protective histones and appears to have less efficient repair mechanisms. Therefore, it is particularly susceptible to damage caused by ROS and other genotoxic agents [4]. Previous studies have indicated a potential involvement of both mtDNA mutations and alterations of mtDNA content (increased or decreased) in the tumorigenesis of many malignancies [5–8]. For example, mtDNA content in patient tissues has been found to be increased in cancers of head and neck, ovary and esophagus [5, 9, 10], and decreased in hepatocellular carcinoma (HCC), advanced gastric cancer, osteosarcoma, breast cancer and renal cell carcinoma (RCC) [8, 11–14]. In addition, several studies have demonstrated that the alterations of mtDNA content in peripheral blood lymphocytes (PBLs) can be used as a surrogate of constitutive genetic background to predict the risk of cancers such as RCC, breast cancer, lung cancer, non-Hodgkin lymphoma (NHL), and colorectal cancer (CRC) [15–19]. However, to date, the association between mtDNA content in PBLs and glioma susceptibility has not been determined.

In the present study, we conducted a case–control epidemiological analysis to examine the association between PBL mtDNA content and glioma risk. We measured the mtDNA content in PBLs from glioma patients and matched healthy controls using quantitative real-time PCR, and evaluated their associations with glioma risk using multivariate logistic regression model. To the best of our knowledge, this is the first epidemiological study to investigate the role of mtDNA content in glioma etiology.

## Methods

### Study population

In this case–control study, patients with histologically confirmed primary glioma were consecutively recruited from the Department of Neurosurgery in Tangdu Hospital affiliated with the Fourth Military Medical University, Xi’an, Shaanxi, China, between February 2010 and June 2012. Among a total of 495 eligible patients, 414 were successfully interviewed and donated biological specimens with a participation rate of 83.6% during the study period. All cases had no previous cancer history and no prior treatment at enrollment. There was no age, sex, or disease stage restriction for case recruitment. The 414 healthy controls without previous cancer history were recruited from individuals who visited the Tangdu Hospital for physical examination, during the same time period as the case enrollment. The response rate of controls was 73.2%. The controls were frequency-matched to the cases on age (±3 years), sex and residential areas. All participants were Han Chinese.

### Epidemiological data

After signed informed consent was obtained from each individual, all participants were interviewed by trained staff interviewers to collect demographic and personal data using a standardized epidemiological questionnaire, including age, gender, smoking history, family history of cancer, ionizing irradiation (IR) exposure history, and other potential confounders. Clinical information on pathological types was collected through pathological reports. Individuals who smoked less than 100 cigarettes during their lifetime were categorized as never-smokers. Individuals who smoked more than 100 cigarettes during their lifetime were categorized as ever-smokers. The number of pack-years was calculated as the average number of cigarettes smoked per day divided by 20 cigarettes and then multiplied by smoking years. All information exhibited high consistency except IR exposure history, which might stem from inaccurate understanding of IR exposure questionnaires. Therefore, data on IR exposure were not used for the further analyses in this study.

Before any treatment, 5 mL of venous blood from each participant was drawn into coded sodium citrate-coated tubes and centrifuged at 4°C under 1200 × g within 30 min. Genomic DNA was extracted from PBLs using the E.Z.N.A. Blood DNA Midi Kit (Omega Bio-Tek, Norcross, GA) and stored at -80°C until PCR examination. Laboratory personnel were blinded to the case–control status of the samples. This study was approved by the Ethical Committee of the Fourth Military Medical University and performed in accordance with the ethical standards of the Helsinki Declaration.

### Determination of mtDNA content by quantitative real-time PCR

Relative mtDNA content was measured by a quantitative real-time PCR-based method as previously described, with the same primers that were used for the mitochondrial ND1 gene (ND-R and ND-F) and the single-copy nuclear gene human globulin (HGB-1and HGB-2) [17]. In short, two pairs of primers were used in the two steps of relative quantification for mtDNA copy number. In the first step, the ratio of mtDNA copy number to HGB copy number was calculated for each sample from standard curves. In the second step, the ratio for each sample was normalized to a calibrator DNA in order to standardize between different runs, and then defined as the measurement of relative mtDNA content.

The PCR reaction system (20 μL) consisted of 1 × SYBR green mastermix (TaKaRa, Dalian, China), 10 nM ND1-R (or HGB-1) primer, 10 nM ND1-F (or HGB-2) primer, and 8 ng of genomic DNA. The thermal cycling conditions for both primer pairs were 95°C for 30 sec, followed by 35 cycles of 94°C for 30 sec, 58°C for 30 sec, and 72°C for 50 sec with signal acquisition. The PCRs were always performed on separate 96-well plates, with the same samples in the same well positions. All samples were assayed in duplicate using the Mx3005P QPCR System (Agilent, Santa Clara, CA). In each run, negative and positive controls, a calibrator DNA, and a standard curve were included. For each standard curve, one reference DNA sample (the same DNA sample for all runs) was diluted with a 3-fold increment per dilution to produce a 5-point standard curve between 0.593 ng and 48 ng DNA in each reaction. The *R*^2^ for each standard curve was ≥ 0.99, with acceptable standard deviations set at 0.25 (for the Ct values). Otherwise, the test was repeated.

### Statistical analysis

All statistical analyses were performed using the SPSS Statistics 19.0 software (IBM). Normally distributed data were expressed as Mean ± SD, while abnormally distributed data were expressed as median with a bracketed range. Pearson χ^2^ test was used to examine differences in the distribution of categorical variables including age, sex, smoking status, and family history of cancer among cases and controls. For the normally distributed continuous variables (pack-years of smoking), Student’s *t* test was used to test the differences between cases and controls. The significance of differences between cases and controls for abnormally distributed continuous variables (mtDNA content) was determined by Mann–Whitney U test. The mtDNA content was also analyzed as a categorical variable by grouping it based on the median, tertile or quartile values in the controls. The association between glioma risk and mtDNA content was estimated using odds ratio (OR) and 95% confidential interval (CI) in unconditional multivariate logistic regression analysis after adjustment by age, sex, smoking status, and family history of cancer, where appropriate. A restricted cubic spline was plotted to evaluate the shape of the association as previously described [20]. Likelihood ratio tests were used to evaluate linear, effect, and overall effects of mtDNA content on glioma risk. All *P* values reported were two-sided, and *P* < 0.05 was considered to be statistically significant.

## Results

A total of 414 glioma patients and 414 matched healthy controls were included in this study. Table 1 summarized the characteristics of each type of distribution. The glioma cases and healthy controls were well-matched on sex (*P* = 1.00) and age (*P* = 0.491). There was no statistically significant difference between cases and controls in terms of family cancer history (*P* = 0.12), smoking status (*P* = 0.108), smoking pack-years (*P* = 0.342), platelet count (*P* = 0.110) white blood cell (WBC) count (*P* = 0.253) or the percentage of neutrophils (*P* = 0.144), lymphocytes (*P* = 0.116) or monocytes (*P* = 0.473) in WBC. Further analysis indicated that no significant correlation was found between mtDNA content and levels of platelet or white blood cell types (data not shown). These data suggest that levels of platelet or white blood cell types may not have notable effect on mtDNA content in blood samples. Among the total 414 cases, 175 patients were diagnosed with low-grade gliomas (WHO grade I/II) and 239 were diagnosed with high-grade gliomas (WHO grade III/IV).

**Table 1:** **Distribution of selected characteristics in glioma cases and healthy controls**

Variables	Case (n = 414)	Control (n = 414)	***P***value
**Age(years), No. (%)**			0.491
<47	204 (49.27)	206 (49.86)	
≥47	210 (50.73)	208 (50.14)	
**Sex, No. (%)**			1.000
Male	241 (58.21)	241 (58.21)	
Female	173 (41.79)	173 (41.79)	
**Smoking status, No. (%)**			0.108
Never	318 (76.81)	329 (79.47)	
Ever	96 (23.19)	85 (20.53)	
**Family history of cancer, No. (%)**			0.103
Yes	45 (10.87)	32 (7.73)	
No	369 (89.13)	382 (92.27)	
**Pack-years of smoking** ^**a**^ **, Mean (SD)**	26.20 (14.94)	25.29 (13.05)	0.342
**White blood cell count (10** ^**9**^ **/L), Mean (SD)**	6.2 (4.17)	5.9 (3.32)	0.253
**% of neutrophils, Mean (SD)**	64.7 (25.32)	62.2 (23.85)	0.144
**% of lymphocytes, Mean (SD)**	28.1 (19.53)	30.3 (20.64)	0.116
**% of monocytes, Mean (SD)**	5.4 (3.88)	5.6 (3.35)	0.473
**Platelet count (10** ^**9**^ **/L), Mean (SD)**	245 (69.05)	253 (74.51)	0.110
**WHO grade**			
I/II	175 (42.27)		
III/IV	239 (57.73)		

SD, standard deviation.

^a^Only for ever smokers.

**Figure 1:**
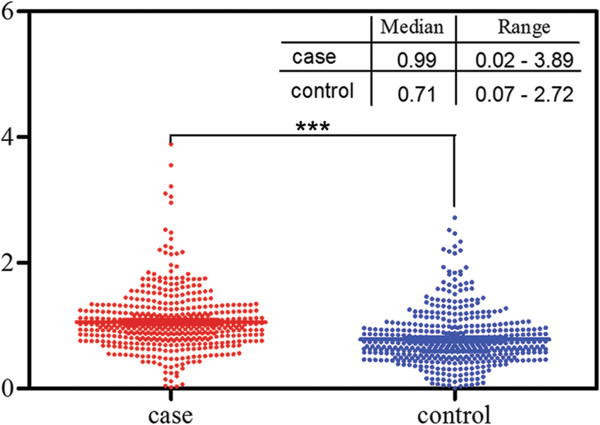
**Comparison of relative mitochondrial DNA (mtDNA) copy number between glioma cases and healthy controls.** Two-sided Mann–Whitney U test was used to evaluate difference of mtDNA copy number between glioma cases and healthy controls. ***, *P* < 0.001.

We measured mtDNA content using a real-time PCR-based method in all samples. The mean inter-assay coefficient variation (CV) of real-time PCR reaction was 6.9% (range, 3.9% to 9.1%), whereas intra-assay CV was 4.2% (range, 2.4% to 6.9%). We observed that mtDNA content in PBLs was significantly higher in glioma cases than that in controls (*P* < 0.001). The median values of normalized mtDNA content were 0.99 (range, 0.02-3.89) and 0.71 (range, 0.07-2.72) in cases and controls, respectively (Figure 1). Furthermore, we compared the mtDNA content according to host characteristics. As shown in Table 2, the case–control difference was still significant in all stratified subgroups. No significant modulating effect of selected characteristics on mtDNA content was found in both cases and controls, with *P* value ranging from 0.101 to 0.982.

**Figure 2:**
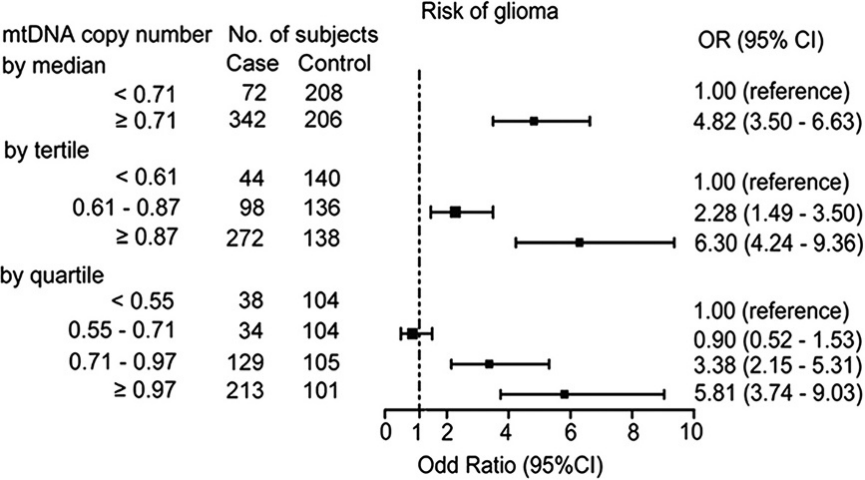
**Risk of glioma as estimated by selected characteristics.** Odds radios (ORs) were calculated by using logistic regression analysis and the tests were two-sided. Both groups were adjusted for age (years, continuous variable), sex, smoking status and family history of cancer where appropriate. Squares indicate study-specific odds ratios; horizontal lines, study-specific confidence intervals (CIs); dotted vertical line, odds ratio of 1.0.

We then performed an unconditional logistic regression analysis to evaluate the association between mtDNA content or other selected characteristics and glioma risk. When participants were dichotomized into high and low groups based on the median value of mtDNA content in controls (Figure 2), we observed that high mtDNA content was significantly associated with a 4.79-fold increase in risk of glioma (95% CI, 3.49-6.59) in the univariate logistic regression model and a 4.82-fold increase in risk of glioma (95% CI, 3.50 - 6.63) after adjusting for the confounding effects of age, sex, smoking status and family history of cancer in the multivariate logistic regression model. Next, participants were categorized into three groups according to the tertile values of mtDNA content in healthy controls (Figure 2). When the first (lowest mtDNA content) tertile was used as the reference group, we observed that the adjusted ORs for the second and third tertile were 2.28 (95% CI, 1.49 - 3.50) and 6.38 (95% CI, 4.24 - 9.36), respectively. When participants were categorized into four groups according to quartile values of mtDNA content in healthy controls, the adjusted ORs for the second, third, and fourth quartiles were 0.90 (95% CI, 0.52 - 1.53), 3.38 (95% CI, 2.15 - 5.31), and 5.81 (95% CI, 3.74 - 9.03), respectively.

**Table 2:** **mtDNA copy number by host characteristics of glioma cases and healthy controls**

Variables	Case		Control		***P***value
	No.	mtDNA copy number, median (range)	No.	mtDNA copy number, median (range)	
**Sex**					
Male	241	0.98 (0.12 to 3.89)	241	0.70 (0.07 to 2.72)	< 0.001
Female	173	1.01 (0.02 to 3.22)	173	0.74 (0.15 to 2.52)	< 0.001
*P* value		0.431		0.101	
**Age, years**					
<47	204	0.96 (0.02 to 3.89)	206	0.70 (0.13 to 2.72)	< 0.001
≥47	210	1.03 (0.03 to 3.55)	208	0.72 (0.07 to 2.52)	< 0.001
*P* value		0.165		0.877	
**Smoking status**					
Never	318	0.98 (0.02 to 3.61)	329	0.69 (0.07 to 2.72)	< 0.001
Ever	96	1.02 (0.03 to3.89)	85	0.71 (0.12 to 2.55)	0.001
*P* value		0.251		0.267	
**Family history of cancer**					
Yes	45	0.90 (0.03 to 3.26)	32	0.72 (0.18 to 2.66)	0.002
No	369	0.99 (0.02 to 3.89)	382	0.69 (0.07 to 2.72)	< 0.001
*P* value		0.151		0.803	
**WHO grade**					
I/II	175	0.98 (0.04 to 3.55)			
III/IV	239	1.00 (0.02 to 3.89)			
*P* value		0.982			

mtDNA, mitochondrial DNA.

**Figure 3:**
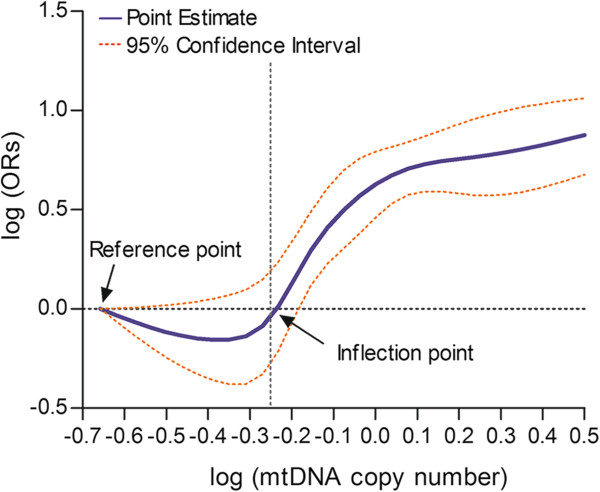
**Association between leukocyte mitochondrial DNA (mtDNA) copy number and subsequent risk of glioma.** mtDNA copy number and odds ratio (OR) values were transformed to common logarithm. There was an S-shaped relationship between mtDNA copy number and glioma risk (*P* for nonlinearity = 0.009).

We further used a restricted cubic spline function in the logistic regression model to evaluate the shape of the association between mtDNA content and glioma risk. As shown in Figure 3, our result exhibited an S-shaped association between them. With the increase of mtDNA content, the glioma risk decreased before the inflection point [log (mtDNA content) = -0.25]; whereas glioma risk gradually increased with the increase of mtDNA content after the inflection point. The *P* value of test for nonlinearity is 0.008. Our stratified analysis showed that higher mtDNA content was associated with increased glioma risk in all strata (Table 3). We also analyzed the interactive effects of mtDNA content and host characteristics on the risk of glioma. The *P* values for the interaction of mtDNA content with sex, age, smoking status and family cancer history were 0.193, 0.467, 0.072 and 0.287, respectively. These data suggest that the association between increased glioma risk and higher mtDNA content was not modulated by major host characteristics.

**Table 3:** **mtDNA copy number and glioma risk estimates by selected variables**

Variables	Cases ^a^(n)		Controls ^a^(n)		Adjusted OR ^b^(95% CI)	***P***value
	High	Low	High	Low		
**Sex**						
Male	198	43	122	119	4.85 (3.52 to 6.71)	<0.001
Female	139	34	86	87	4.26 (2.83 to 5.94)	<0.001
**Age, years**						
<47	167	37	103	103	4.81 (3.42 to 6.69)	<0.001
≥47	170	40	105	103	4.84 (3.59 to 6.65)	<0.001
**Smoking status**						
Never	275	43	166	163	4.92 (3.43 to 6.82)	<0.001
Ever	62	34	42	43	1.98 (1.14 to 3.02)	0.014
**Family history of cancer**						
Yes	28	17	16	16	2.14 (1.19 to 3.36)	0.006
No	309	60	192	190	4.89 (3.36 to 6.84)	<0.001

95% CI, 95% confidence interval; mtDNA, mitochondrial DNA; OR, odds ratio.

^a^Cases and controls were dichotomized based on the median value of controls.

^b^Adjusted by age, sex, smoking status, family history of cancer and histology type where appropriate.

## Discussion

In this case–control study, we found that glioma patients exhibited significantly higher mtDNA content than healthy controls. Our findings also demonstrated a typical S-shaped association between high mtDNA content and increased glioma risk. These results suggest that mtDNA content in PBLs might be a potential susceptibility biomarker for early preventive screening of glioma. To date, there are only a few risk factors identified to be associated with the risk of glioma, which only account for a small part of glioma cases [21]. Therefore, if our data are confirmed, novel strategy based on leukocyte mtDNA content examination can be established and would help to improve the screening of individuals who would probably develop glioma.

Several previous studies reported that higher mtDNA content in PBLs was significantly associated with an increased risk of NHL, lung cancer, and breast cancer [18, 19, 22]. These results are consistent with our present finding, indicating for the first time a similar positive correlation between PBL mtDNA content and glioma risk. Moreover, significant increase in mtDNA content has been found in both malignant glioma cell lines and tissues, suggesting that mtDNA content alteration may be an early molecular event in the development and progression of glioma [23–25]. Previous studies have also yielded similar results in cancers of endometrium, head and neck, thyroid gland [26], ovary [9], large intestine [27, 28], and lung [27], where mtDNA content was significantly higher in tumor tissues as compared with the corresponding non-tumor adjacent tissues. However, on the contrary, previous studies have also reported negative correlations between mtDNA content and risk of cancers such as HCC [29] and RCC [17]. Furthermore, in comparison to paired normal tissue, a significant decrease in mtDNA content was reported in the tumor tissue of cancers including HCC [29], gastric carcinoma [11], breast cancer [13, 30], and RCC. Therefore, it is most likely that the change in mtDNA content is not simply a function of enhanced cellular proliferation in neoplastic cells, but also has some degree of specificity for particular cancer type. The reason for the tumor-specific association between mtDNA content and cancer risk remains to be evaluated, although it is likely to be regulated by various genetic, molecular, and cellular determinants. Further studies are needed to elucidate the molecular mechanisms underlying the association between mtDNA content and cancer risk.

In our study, we found that glioma cases exhibited higher leukocyte mtDNA content than healthy controls. However, this observational study could not tell whether mtDNA content alterations are the cause or consequence of tumorigenesis, which is a limitation inherent in case–control study design. A previous study have reported that mtDNA content appears to have high heritability (ie, proportion of phenotypic variation in a population that is attributable to genetic variation among individuals) [17]. In addition, several prospective studies have demonstrated that higher mtDNA content is associated with the risks of CRC [31], NHL [19], pancreatic cancer [32] and lung cancer [18]. In addition, our data also showed that glioma grade did not exhibit any remarkable effect on mtDNA content. All these findings suggest that alterations of mtDNA content may happen before cancer establishment. Future studies including animal cancer models and large prospective cohorts are needed to investigate the mtDNA content alterations and its biological roles in glioma.

Considering the crucial role of oxidative stress in tumorigenesis of glioma [25], our finding that higher mtDNA content was associated with an increased risk of glioma is not surprising. Elevated mtDNA content is commonly caused by some forms of oxidative stress in experimental models [33–35]. It has been shown that cells under mild oxidative stress may increase biogenesis of mitochondria and mtDNA through a pathway that bypasses cell-cycle control [36]. During the process of ROS-associated oxidative phosphorylation, accumulation of mtDNA mutations may occur [37]. Furthermore, mutated mtDNA may lead to aberrant mitochondrial biogenesis and then confer a replicative advantage to the cells [38]. Therefore, in our study, the case–control difference in mtDNA content might reflect the possible case–control difference in oxidative stress.

In the present study, we did not find any significant association between the mtDNA content and major host characteristics such as sex, age, smoking, and family history of cancer in both cases and controls. These observations are in line with some of the previous reports, but inconsistent with others that showed age- or smoking-dependent changes in mtDNA content [39, 40]. Additional larger studies with greater statistical power are needed to provide additional insights into the effects of interaction between mtDNA content and host variables on the modulation of glioma risk.

This study has several strengths and limitations. Our population is enrolled from Xi’an and its adjacent areas, which are highly attractive for conducting population-based research. The geographical stability with low mobility rate could greatly reduce the potential confounding effects of the heterogeneous participant characteristics. However, because it was not a random sample of the general population, there was still a certain risk of selection bias if there were any difference in terms of the studied exposures. Moreover, due to inaccurate understanding of IR exposure questionnaire by participants, IR exposure data was not acceptably consistent when cross-check was performed by independent interviewers. We thus were unable to evaluate the mtDNA-IR interactions underlying risk of gliomas. Because the frequency of IR was rather low as reported by epidemiological studies in China [41], studies with larger sample size are still needed for a meaningful analysis on this interaction in future. In addition, our study cannot bypass the reverse-causation problem, an intrinsic limitation of the case–control study design, although previous studies have provided strong direct evidence that mtDNA content may serve as a constitutive genetic marker for cancer susceptibility. Therefore, future prospective epidemiological studies are warranted to further confirm our findings.

## Conclusions

In summary, our data for the first time demonstrated that higher mtDNA content in PBLs was significantly associated with increased glioma risk. This is an initial step to evaluate whether the mtDNA content measured in PBLs can be used as a biomarker for early preventive screening of glioma. Once validated, mtDNA content could be incorporated with other available risk factors to construct a multivariate risk assessment model for identifying subjects with high risk of glioma.
